# Supplementary material for: Chemical Authentication and Speciation of Salvia Botanicals: An Investigation Utilizing GC/Q-ToF and Chemometrics
Source: Foods. 2022 Jul 19;11(14):2132. doi: 10.3390/foods11142132 (PMC9322183; doi:10.3390/foods11142132)
Supplement: Supplementary file 1 [file foods-11-02132-s001.zip › foods-1794659-supplementary.pdf]

Chemical Authentication and Speciation of Salvia Botanicals: An Investigation Utilizing GC/Q-ToF and Chemometrics

Joseph Lee[1], Mei Wang[2*], Jianping Zhao[1], Bharathi Avula[1], Amar G. Chittiboyina[1], Jing Li[4], Charles Wu[4] and Ikhlas A. Khan[1,3,*]

[1] National Center for Natural Products Research, School of Pharmacy, University of Mississippi, University, MS 38677, United States

[2] Natural Products Utilization Research Unit, Agricultural Research Service, United States Department of Agriculture, University, MS 38677, United States

[3] Division of Pharmacognosy, Department of BioMolecular Sciences, School of Pharmacy, University of Mississippi, University, MS 38677, USA

[4] Botanical Review Team, Office of New Drug Product, Office of Pharmaceutical Quality, Center for Drug Evaluation and Research, Food and Drug Administration, 10903 New Hampshire Ave, Silver Spring, MD 20993

Corresponding Author

*Dr. Mei Wang or Dr. Ikhlas A. Khan

Tel: +1 662-915-1046 or +1 662-915-7821

E-mail: meiwang@usda.gov or ikhan@olemiss.edu

Supplemental Material

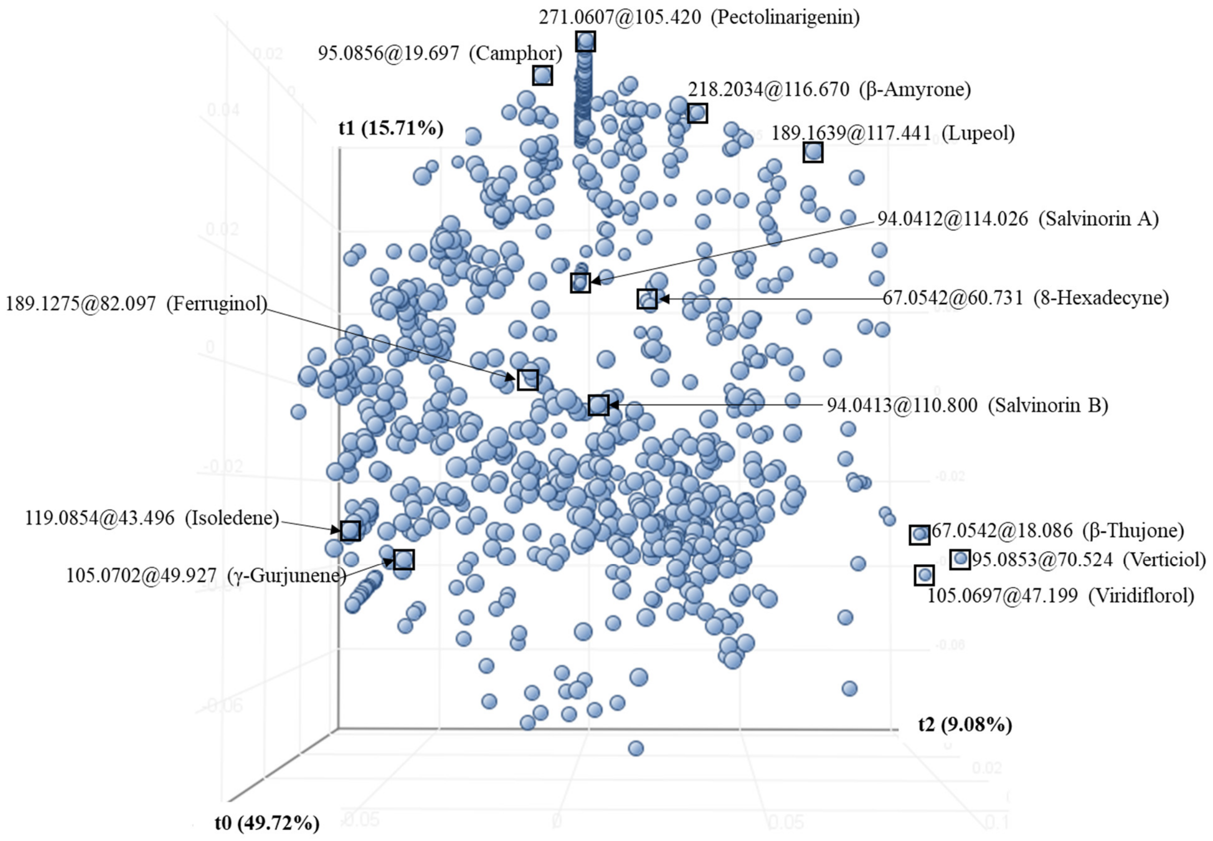

Figure S1. PCA loading plot illustrating suggested species marker compounds.
